# Usefulness of pre-thyroidectomy neutrophil–lymphocyte, platelet–lymphocyte, and monocyte–lymphocyte ratios for discriminating lymph node and distant metastases in differentiated thyroid cancer

**DOI:** 10.6061/clinics/2021/e3022

**Published:** 2021-08-05

**Authors:** Cínthia Minatel Riguetto, Icléia Siqueira Barreto, Frederico Fernandes Ribeiro Maia, Lígia Vera Montali da Assumpção, Denise Engelbrecht Zantut-Wittmann

**Affiliations:** I Divisao de Endocrinologia, Departamento de Medicina Interna, Faculdade de Ciencias Medicas, Universidade Estadual de Campinas, Campinas, SP, BR; II Departamento de Patologia, Faculdade de Ciencias Medicas, Universidade Estadual de Campinas, Campinas, SP, BR

**Keywords:** Neutrophil-Lymphocyte Ratio, Platelet-Lymphocyte Ratio, Monocyte-Lymphocyte Ratio, Distant Metastasis, Differentiated Thyroid Cancer

## Abstract

**OBJECTIVE:**

This study aimed to analyze the relationship of neutrophil-lymphocyte ratio (NLR), platelet-lymphocyte ratio (PLR), and monocyte-lymphocyte ratio (MLR) with clinicopathological characteristics of patients with differentiated thyroid cancer (DTC).

**METHODS:**

This retrospective study included 390 patients with DTC who had complete blood cell counts available at the time of surgery. NLR, PLR, and MLR were calculated, and the risk of cancer-related death, structural recurrence, and response to therapy were assessed using the eighth edition of the tumor-node-metastasis classification, American Thyroid Association (ATA) Risk Stratification System, and ATA Response to Therapy Reclassification, respectively.

**RESULTS:**

PLR was higher in patients with distant metastasis than in those without (133.15±43.95 *versus* 119.24±45.69, *p*=0.0345) and lower in patients with disease-free status (117.72±44.70 *versus* 131.07±47.85, *p*=0.0089) than in those who experienced persistent disease or death. Patients aged ≥55 years had a higher MLR than those aged <55 years (0.26±0.10 *versus* 0.24±0.12, *p*=0.0379). Higher MLR (odds ratio [OR]: 8.775, 95% confidence interval [CI]: 1.532-50.273, *p*=0.0147), intermediate ATA risk (OR: 4.892, 95% CI: 2.492-9.605, *p*≤0.0001), and high ATA risk (OR: 5.998, 95% CI: 3.126-11.505, *p*≤0.0001) were risk factors associated with active disease. NLR was not significantly different among the studied variables. Receiver operating characteristic curve cut-off values for NLR, PLR, and MLR were able to differentiate distant metastasis from lymph node metastasis (NLR>1.93: 73.3% sensitivity and 58.7% specificity, PLR>124.34: 86.7% sensitivity and 69.2% specificity, MLR>0.21: 80% sensitivity and 45.2% specificity).

**CONCLUSION:**

Cut-off values of NLR, PLR, and MLR differentiated distant metastasis from lymph node metastasis with good sensitivity and accuracy. PLR was associated with disease-free status and it was higher in DTC patients with distant metastasis, persistent disease, and disease-related death. MLR was a risk factor for active disease.

## INTRODUCTION

Cancer-associated inflammation plays an essential role in the clinical evolution of most of the cancers, mainly by promoting tumor cell proliferation, angiogenesis, invasion, and metastasis (1-3). Increasing evidence has shown that several biomarkers related to inflammation such as C-reactive protein levels, neutrophil-lymphocyte ratio (NLR), platelet-lymphocyte ratio (PLR), and monocyte-lymphocyte ratio (MLR) or lymphocyte-monocyte ratio (LMR) are indicators of poor prognosis in a variety of cancers (4) and chronic diseases (5-7). However, the importance of such an inflammatory response is not entirely clear in thyroid cancer. NLR is the most widely investigated inflammatory marker among patients with thyroid cancer, and many authors have demonstrated that a high NLR is closely related to aggressive tumors, lymph node metastasis, and poor prognosis (8-13). Similarly, a high PLR is also linked to poor prognosis, especially among patients with papillary and medullary thyroid cancer (14-16). Recently, some authors (17-20) have demonstrated that a low LMR could predict recurrence and overall survival in patients with papillary thyroid cancer (PTC) and poor survival in patients with anaplastic thyroid carcinoma. To the best of our knowledge, no study has evaluated NLR, PLR, and MLR obtained from patients with differentiated thyroid carcinoma (DTC) in addition to their relationship with different histological types and long-term clinical and laboratory outcomes.

The present study aimed to analyze the potential relationship of NLR, PLR, and MLR with clinicopathological characteristics and outcomes in patients with DTC.

## MATERIALS AND METHODS

### Subjects

This retrospective study involved 1,143 patients diagnosed with DTC who were followed-up at the Thyroid Disease Unit at the Clinics Hospital of the University of Campinas between January 2000 and January 2019. Ethical committee approval for the study was obtained according to the Declaration of Helsinki from the Human Research Ethics Committee in Lausanne (No. 204/14. CAAE: 58234916.3.0000.5404).

**Figure 1:**
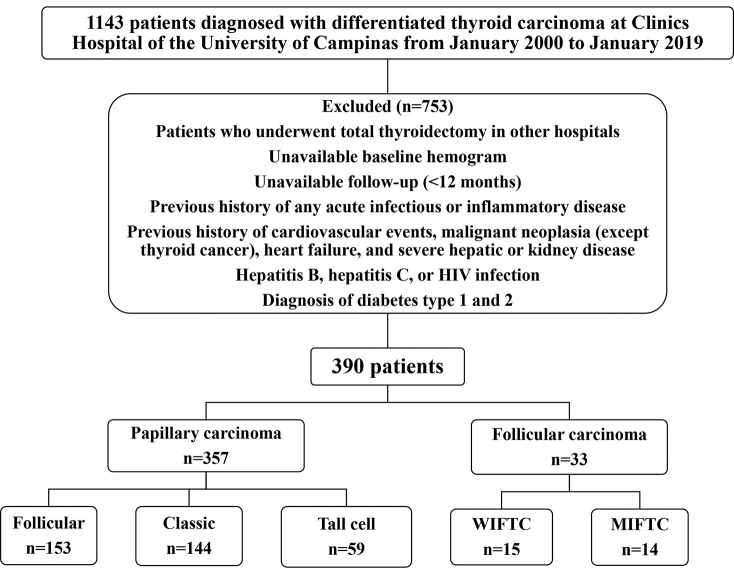
Study flowchart. WIFTC: widely invasive follicular thyroid carcinoma, MIFTC: minimally invasive follicular thyroid carcinoma, HIV: human immunodeficiency virus.

Among the 1,143 initially selected patients, 390 had adequate clinicopathological information and were followed-up. Seven hundred and fifty-three patients were excluded from the study for the following reasons: underwent total thyroidectomy in other hospitals, unavailability of baseline hemogram before the surgery, and inadequate follow-up (a minimum of 12-month follow-up was required). Patients with a previous history of any acute infectious or inflammatory diseases; history of cardiovascular events (myocardial ischemia, unstable angina, or stroke); malignant neoplasia (except thyroid cancer); heart failure (New York Heart Association class III or IV); severe hepatic or kidney disease (chronic kidney disease stages 4 or 5 or hemodialysis); hepatitis B, hepatitis C, and human immunodeficiency virus infection; and diabetes (type 1 and 2) were also excluded. Figure 1 shows the flowchart of the study.

### Clinical assessment

Clinical characteristics and laboratory data were collected through chart review. The collected clinical data included sex, age, age at diagnosis, ethnicity, smoking habit, duration of follow-up, use of ablative and/or adjuvant radioactive iodine therapy, findings from radioactive iodine whole-body scanning, cervical ultrasound, and computed tomography scan when available, and response to cancer treatment.

Tumor characteristics analyzed in the study included histology (papillary or follicular), PTC variants (classic, follicular, or tall cell), tumor size (largest dimension of the lesion), multifocality, invasion (extrathyroidal extension or capsular or vascular invasion), metastatic nodes, and distant metastasis.

Laboratory data included serum levels of thyroid-stimulating hormone (reference value 0.41-4.5 mUI/L), free thyroxine (reference value 0.9-1.8 m/dL), thyroglobulin antibodies (reference value <115 mUI/L), and thyroglobulin (reference value 0.2-70 ng/mL) measured using electrochemiluminescence immunoassay. Complete blood counts and automated differential counts were collected 3 days before the surgery. NLR, PLR, and MLR were calculated from the complete blood. NLR was calculated by dividing the number of neutrophils by the number of lymphocytes. Similarly, PLR and MLR were calculated by dividing the number of platelets and monocytes by the lymphocyte count, respectively.

### Risk stratification

The eighth edition of the tumor-node-metastasis (TNM) classification developed by the American Joint Committee on Cancer was used to classify thyroid tumors and to predict the risk of cancer-related death. The risk of structural recurrence was assessed using the American Thyroid Association (ATA) risk stratification system, based on which the risk can be classified as low, intermediate, or high. Response to treatment was assessed using the ATA Response to Therapy Reclassification, and the response was classified as excellent, biochemical incomplete, structural incomplete, or indeterminate. Disease outcomes were classified as disease-free status, persistent disease, or disease-related death.

### Statistical analysis

Statistical analyses were performed using SAS for Windows, version 9.4 (SAS Institute Inc., Cary, NC, USA). Exploratory data analysis was performed using summary measures (mean, standard deviation, minimum, median, maximum, frequency, and percentage). The relationship of NLR, PLR, and MLR with the variables of interest was assessed using Mann-Whitney or Kruskal-Wallis tests. Univariate and multiple Cox regression analyses were used to identify the factors associated with disease-free status. Stepwise variable selection process was employed. The receiver operating characteristic (ROC) curve was used to assess the predictive accuracy of the ratios. Larger area under the ROC curve (AUC) indicated greater discriminatory power of the model. Generally, an AUC≥0.7 indicates acceptable discriminatory power of the model. The significance level was set at *p*<0.05.

## RESULTS

### Baseline characteristics

Table 1 shows the detailed characteristics of 390 patients with DTC who were included in this study. Most of the included patients were female (83.08%), Caucasians (73.08%), and non-smokers (70.51%). The mean age of the patients was 46.65±15.41 years. The majority of the patients were diagnosed with PTC (91.54%) and were classified as either classic (40.45%), follicular (42.98%), or tall cell (16.57%) variants. Based on the ATA risk stratification system, 50% of the patients were classified as low-risk, 25.90% as intermediate-risk, and 24.10% as high-risk patients. Despite the high proportion of patients with intermediate and high risk, most of them (78.72%) presented an excellent response to treatment. The rates of structural incomplete response, indeterminate response, and biochemical incomplete response were 8.21%, 7.95%, and 5.13%, respectively. Our classification of disease outcomes into disease-free status (78.72%), persistent disease (19.49%), and disease-related death (1.79%) was similar to the ATA Response to Therapy Reclassification. The mean NLR, PLR, and MLR were 2.05±1.12, 120.56±45.65, and 0.25±0.11, respectively.

### Relationship of NLR, PLR, and MLR with tumor characteristics, stage, and evolution

Table 2 shows the comparison between NLR, PLR, and MLR with patient’s age, histology, PTC variants, tumor size, type of invasion, focal and distant metastasis, and risk stratification. NLR was not statistically significant in any variable analyzed. In contrast, PLR was higher among patients with distant metastasis than in those without it (133.15±43.95 *versus* 119.24±45.69, *p*=0.0345). PLR was significantly lower in patients classified as disease-free status than in those with persistent disease or disease-related death (117.72±44.70 *versus* 131.07±47.85, *p*=0.0089). Patients aged ≥55 years exhibited a significantly higher MLR than those aged <55 years (0.26±0.10 *versus* 0.24±0.12, *p*=0.0379).

### Cox univariate and multiple logistic regression analyses regarding disease-free status

In the univariate logistic regression analysis, a higher PLR (odds ratio [OR]: 1.004, 95% confidence interval [CI]: 1.001-1.008, *p*=0.0247), tumor size of ≥1 cm (OR: 2.942, 95% CI: 1.416-6.113, *p*=0.0038), age greater than 55 years (OR: 1.701, 95% CI: 1.096-2.640, *p*=0.0179), TNM stages III and IVb *versus* stages I and II (OR: 3.268, 95% CI: 1.982-5.389, *p*≤0.0001), tall cell variant *versus* follicular variant (OR: 2.016, 95% CI: 1.106-3.676, *p*=0.0221), intermediate (OR: 4.916, 95% CI: 2.509-9.631, *p*≤0.0001), and high ATA risk *versus* low risk (OR: 6.652, 95% CI: 3.583-12.352, *p*≤0.0001) were factors significantly associated with a low rate of disease-free status (Table 3).

A multiple logistic regression analysis was performed with a model including the following variables used in the univariate analysis: age≥55 years *versus* <55 years, NLR, MLR, PLR, tumor size of ≥1 cm *versus* <1 cm, TNM stages III and IV *versus* stages I and II, papillary carcinoma variant (tall cells *versus* follicular, classic *versus* follicular), and ATA risk (high *versus* low, intermediate *versus* low). A higher MLR (OR: 8.775, 95% CI: 1.532-50.273, *p*=0.0147), intermediate ATA risk (OR: 4.892, 95% CI: 2.492-9.605, *p*≤0.0001), and high ATA risk (OR: 5.998, 95% CI: 3.126-11.505, *p*≤0.0001) were factors associated with a lower disease-free status (Table 3).

### ROC curve analysis

**Figure 2:**
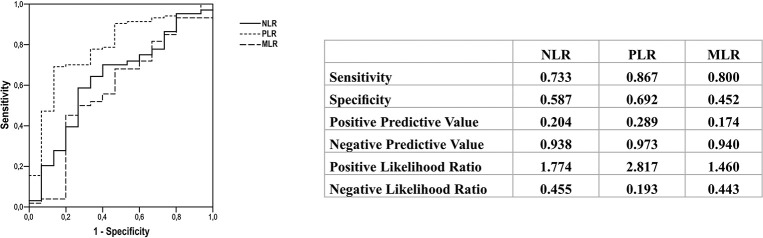
Receiver operating characteristic curves showing the cut-off values of neutrophil-lymphocyte ratio (NLR), platelet-lymphocyte ratio (PLR), and monocyte-lymphocyte ratio (MLR) for discriminating distant metastasis from lymph node metastasis.

ROC curves allowed the identification of discriminatory cut-off values for each analysis. Values above the cut-off were observed among patients with distant metastasis and not in those with lymph node metastasis. The best cut-off values to differentiate lymph node metastasis from distant metastasis were as follows: NLR>1.93 (sensitivity of 73.3%, specificity of 58.7%, accuracy of 63.5%, positive predictive value of 20.4%, and negative predictive value of 93.8%), PLR>124.34 (sensitivity of 86.7%, specificity of 69.2%, accuracy of 78.7%, positive predictive value of 28.9%, and negative predictive value of 97.3%), and MLR>0.21 (sensitivity of 80%, specificity of 45.2%, accuracy of 57.9%, positive predictive value of 17.4%, and negative predictive value of 94%) (Figure 2). These parameters did not allow discrimination between distant metastasis and disease-free status (AUC: NLR, 0.574; PLR, 0.575; and MLR, 0.550), between lymph node metastasis and disease-free status (AUC: NLR, 0.540; PLR, 0.544; and MLR, 0.540), between metastasis (lymph node and distant) and no metastasis (AUC: NLR, 0.524; PLR, 0.514; and MLR, 0.502), and between metastasis (lymph node and distant) and disease-free status (AUC: NLR, 0.552; PLR, 0.581; and MLR, 0.550) (data not shown).

## DISCUSSION

This study evaluated the role of NLR, PLR, and MLR as predictive tools for disease status in patients with DTC. The cut-off values of NLR, PLR, and MLR enabled the discrimination of distant metastasis from lymph node metastasis with good sensitivity and accuracy. In addition, we demonstrated that MLR associated with a low ATA risk in patients with DTC was a predictive factor of disease-free status. Furthermore, PLR was related to disease-free status, and it was comparatively higher in DTC patients with distant metastasis, persistent disease, and disease-related death than in patients free of disease. However, we did not find any association between NLR and disease outcome in the same population.

NLR, PLR, and MLR may reflect two connected processes, namely systemic inflammatory response and immune system status. The exact pathways between these ratios and tumor behavior remain unclear. However, it is well known that cancer cells can secrete inflammatory factors such as interleukin (IL)-1, IL-2, IL-6, IL-8, IL-12, IL-17, granulocyte colony-stimulating factor, and tumor necrosis factor-alpha, which may contribute to the tumor microenvironment, leading to tumor invasion, leukocytosis, and thrombocytosis (4,21). Neutrophilia, thrombocytosis, and increased monocytes may be observed in tissue damage and inflammation, playing an essential role in tumor proliferation, invasion, metastasis, and recurrence. Lymphocytes are the leading components of immunity against tumors, stimulating the release of cytokines such as interferons and tumor necrosis factor-alpha, which results in subsequent tumor reduction. Decreased lymphocyte count is associated with an inadequate immunologic reaction that increases the susceptibility to tumor progression and metastasis (1,2,4,22).

NLR is the most widely investigated marker in thyroid cancer as well as in several other malignancies including gastrointestinal tumors and breast, lung, prostate, and ovarian cancers (4). Most studies that analyzed NLR in patients with DTC have shown that a high NLR was associated with poor prognosis and survival, and it was primarily related to TNM classification, patients’ age, tumor size, multifocality, lymph node metastasis, extrathyroidal invasion, and risk of recurrence (8-13). However, two meta-analyses differ in their results regarding the utility of NLR (23,24). Feng et al. (23) suggested that NLR may serve as a biomarker to predict tumor growth, metastasis, and prognosis, whereas Liu et al. (24) stated that NLR is not a reliable indicator of prognosis in patients with DTC. Consistent with the latter, our study could not confirm the usefulness of NLR since no association was found between NLR and prognosis or tumor features in the included patients. In contrast, we could determine a cut-off value of NLR through the ROC curve wherein NLR>1.93 discriminated distant metastasis from lymph node metastasis (sensitivity of 73.3% and accuracy of 63.5%).

Although PLR was assessed less frequently than NLR in previous studies, some authors have found a link between PLR and poor prognosis among patients with DTC (13,16,21). We showed that patients with distant metastasis presented a higher PLR, as well as a lower PLR was found in patients classified as disease-free status. Besides that, PLR>124 helped differentiate distant metastasis from lymph node metastasis with high sensitivity (86.7%) and accuracy (78.7%). The majority of the studies have analyzed patients with DTC, however, two Chinese studies suggested that a high PLR was associated with poor prognosis in medullary thyroid cancer, while NLR did not exhibit such an association (14,15). Jiang et al. (14) demonstrated an association of high PLR with lymph node metastasis and recurrence. A year later, the same group reported that increased PLR was also predictive of lymph node metastasis, capsular invasion, advanced tumor stages, and recurrence (15).

Very few studies have evaluated MLR or LMR in patients with thyroid cancer (17-20,25). Ahn et al. (19) performed a retrospective study including 35 patients with anaplastic thyroid cancer and found an association between low LMR and poor survival. In contrast, Yamazaki et al. (25) evaluated a greater number of patients with anaplastic thyroid cancer and did not find a similar association. Three other authors (17,18,20) assessed LMR in patients with PTC and found that a low preoperative LMR could predict recurrence and overall survival. Although we assessed the inverse ratio (MLR), a similar relationship was seen in our study. We observed a higher MLR among older patients (≥55 years old) and it was a predictive factor for disease-free status. Additionally, we found that an MLR of <0.21 could discriminate distant metastasis from lymph node metastasis, with a sensibility of 80% and an accuracy of 58%.

A small number of studies have demonstrated the relationship between these ratios and benign thyroid diseases such as Graves’ disease (GD), Hashimoto’s thyroiditis (HT), toxic adenoma (TA), and subacute thyroiditis (SAT) (26-30). Hu et al. (28) proposed combining thyroid hormones (free thyroxine and triiodothyronine) and the eosinophil-to-monocyte ratio to distinguish GD from SAT. Taskaldiran et al. (30) retrospectively analyzed NLR and PLR in patients with GD, SAT, and TA and suggested that high PLR and NLR may be useful in differentiating SAT from GD and TA. Another interesting retrospective study performed by Kim et al. (29) reported that elevated NLR was an independent prognostic factor for relapse in patients with GD. Aktas et al. (26) and Bilge et al. (27) found a significantly higher NLR in patients with HT than in healthy controls.

This study had some limitations including those inherent to a retrospective and single-institution study. Moreover, the total number of subjects included in this study was small compared to other studies; however, our analysis was able to present robust results. Despite these limitations, the present study is the first to analyze three different ratios covering the low, intermediate, and high risk of metastasis among patients with DTC.

## CONCLUSION

We found that cut-off values of NLR, PLR, and MLR were able to discriminate distant metastasis from lymph node metastasis with good sensitivity and accuracy. High PLR and MLR were associated with active disease status. Lower PLR was associated with disease-free status, while a higher PLR was observed in DTC patients with distant metastasis, persistent disease, and disease-related death. Our study demonstrated that PLR, NLR, and MLR could be considered useful, readily available, and low-cost tools during clinical follow-up of patients with DTC. However, a large-scale study is required to confirm our results.

## AUTHOR CONTRIBUTIONS

Riguetto CM, Barreto IS, Maia FFR, Assumpção LVM and Zantut-Wittmann DE participated in the data acquisition, analysis and interpretation. Riguetto CM and Wittmann DEZ participated in the conception and design of the study, interpretation of results, and manuscript drafting.

## Figures and Tables

**Table 1:** Baseline and histopathological characteristics and outcomes of patients with differentiated thyroid carcinoma.

Patients’ characteristics		n=390
Gender	Female	324 (83.08%)
	Male	66 (16.92%)
Age (years)		46.65±15.41
Age	<55 years	262 (67.18%)
	≥55 years	128 (32.82%)
Follow-up (months)		82.83±54.17
Platelets (×10^3^/µL)		250±63
White blood cells (×10^3^/µL)		7.20±1.91
Neutrophils (×10^3^/µL)		4.16±1.48
Lymphocytes (×10^3^/µL)		2.26±0.76
Monocytes (×10^3^/µL)		0.52±0.20
Neutrophil-lymphocyte ratio		2.05±1.12
Platelet-lymphocyte ratio		120.56±45.65
Monocyte-lymphocyte ratio		0.25±0.11
DTC characteristics		n=390
Thyroid cancer	Papillary	357 (91.54%)
	Follicular	33 (8.46%)
Papillary thyroid cancer variants	Classic	144 (40.45%)
	Follicular	153 (42.98%)
	Tall cell	59 (16.57%)
Diameter of the largest tumor focus (cm)		2.31±1.96
Microcarcinoma		101 (25.96%)
Multifocality		202 (52.06%)
Invasion	Extrathyroidal extension	92 (23.71%)
	Capsular	87 (22.42%)
	Vascular	83 (21.39%)
	No invasion	126 (32.48%)
Metastatic lymph nodes		126 (32.31%)
Distant metastasis	Lung	29 (7.44%)
	Bone	4 (1.03%)
	Lung and bone	4 (1.03%)
T1		181 (46.53%)
T2		69 (17.74%)
T3		86 (22.11%)
T4		53 (13.62%)
N0		263 (67.61%)
N1		116 (32.39%)
M0		351 (90.23%)
M1		38 (9.77%)
TNM staging (eighth edition)	I	296 (76.09%)
	II	58 (14.91%)
	III	16 (4.11%)
	IV	19 (4.88%)
ATA risk	Low	195 (50%)
	Intermediate	101 (25.90%)
	High	94 (24.10%)
Radioiodine therapy		366 (94.33%)
Radioiodine activities		195.16±198.34
Anti-thyroglobulin antibody detectable		17 (4.36%)
Response to treatment	Excellent	307 (78.72%)
	Biochemical incomplete	20 (5.13%)
	Structural incomplete	32 (8.21%)
	Indeterminate	31 (7.95%)
Tumor outcome	Disease-free status	307 (78.72%)
	Persistent disease	76 (19.49%)
	Disease-related death	7 (1.79%)

Values are presented as mean±standard deviation or as count (percentage).

TNM: tumor-nodes-metastasis, ATA: American Thyroid Association, DTC: differentiated thyroid cancer.

**Table 2:** Association of neutrophil-lymphocyte ratio, platelet-lymphocyte ratio, and monocyte-lymphocyte ratio with tumor characteristics, stage, and evolution of differentiated thyroid carcinoma.

	Patients	Neutrophil-lymphocyte ratio		Platelet-lymphocyte ratio		Monocyte-lymphocyte ratio	
		Mean	*p*-value	Mean	*p*-value	Mean	*p*-value
Thyroid Cancer							
Papillary	357	2.05±1.15	0.4491	120.11±44.66	0.7714	0.25±0.12	0.2949
Follicular	33	2.04±0.73		125.45±55.89		0.25±0.09	
Variants							
Classic PTC	144	2.04±1.18	0.8484	118.76±39.65	0.9810	0.23±0.09	0.6133
Follicular	153	2.06±1.22		120.98±48.76		0.26±0.13	
Tall cell	59	2.01±0.87		120.74±46		0.26±0.14	
Capsular invasion							
Yes	87	1.91±0.74	0.6555	119.16±45.91	0.7353	0.25±0.12	0.8818
No	301	2.08±1.20		120.81±45.76		0.25±0.11	
Angiolymphatic invasion							
Yes	83	2.06±1.15	0.7962	120.39±41.86	0.6194	0.25±0.12	0.5674
No	305	2.04±1.11		120.45±46.81		0.25±0.11	
Extrathyroidal invasion							
Yes	92	2.06±1.19	0.8269	118.81±41.33	0.9478	0.25±0.13	0.9550
No	296	2.04±1.10		120.95±47.08		0.25±0.11	
Lymph node metastasis							
Yes	126	2.04±1.08	0.9334	115.58±38.14	0.3197	0.24±0.11	0.7524
No	264	2.05±1.14		122.94±48.72		0.25±0.12	
Distant metastasis							
Yes	37	2.15±0.91	0.1786	133.15±43.95	**0.0345**	0.24±0.08	0.4285
No	353	2.03±1.14		119.24±45.69		0.25±0.12	
Tumor Size							
<1 cm	288	2.03±1.04	0.9992	118.38±41.74	0.7888	0.25±0.10	0.4843
≥1 cm	101	2.05±1.15		121.25±47.05		0.25±0.12	
Age							
<55 years	262	2.04±1.17	0.3177	121.17±44.69	0.3688	0.24 ± 0.12	**0.0379**
≥55 years	128	2.06±1.00		119.31±47.72		0.26 ± 0.10	
TNM staging (eighth edition)							
Stages I and II	354	2.04±1.15	0.4112	119.20±45	0.1004	0.25±0.12	0.0986
Stages III and IV	35	2.06±0.8		132.64±50.53		0.26±0.10	
ATA risk							
Low	195	2.09±1.22	0.8286	121.85±48.66	0.4852	0.25±0.12	0.4317
Intermediate	101	1.98±0.96		115.44±40.44		0.24±0.13	
High	94	2.09±1.22		121.85±48.66		0.25±0.12	
Radioiodine therapy							
Yes	361	2.06±1.13	0.3018	120.46±45.50	0.8120	0.25±0.12	0.8856
No	32	1.80±0.84		118.47±49.47		0.24±0.08	
Response to treatment							
Excellent	307	2.01±1.11	0.3453	117.72±44.70	0.0717	0.24±0.11	0.4423
Biochemical incomplete	20	2.27±1.30		128.54±44.41		0.27±0.12	
Structural incomplete	32	2.05±0.77		130.46±49.54		0.25±0.10	
Indeterminate	31	2.25±1.36		133.34±49.64		0.27±0.16	
Disease outcome							
Disease-free status	307	2.01±1.11	0.0812	117.72±44.70	**0.0089**	0.24±0.11	0.1219
Persistent disease and disease-related death	83	2.18±1.14		131.07±47.85		0.26±0.13	

Values are presented as mean±standard deviation. Statistical significance was set at *p*<0.05.

PTC: papillary thyroid cancer, TNM: tumor-node-metastasis, ATA: American Thyroid Association.

**Table 3:** Factors associated with disease-free status in patients with differentiated thyroid cancer.

Variables	*p*-value	HR	95% CI
Cox univariate analysis			
NLR	0.4074	1.072	0.909-1.265
PLR	0.0247	1.004	1.001-1.008
MLR	0.0904	4.272	0.796-22.944
Tumor size of ≥1 cm *versus* <1 cm	0.0038	2.942	1.416-6.113
Age≥55 years *versus* <55 years	0.0179	1.701	1.096-2.640
TNM stages III and IV *versus* I and II	<0.0001	3.268	1.982-5.389
Papillary variant			
Tall cell *versus* follicular	0.0221	2.016	1.106-3.676
Classic *versus* follicular	0.9494	1.018	0.590-1.756
High *versus* low ATA risk	<0.0001	6.652	3.583-12.352
Intermediate *versus* low ATA risk	<0.0001	4.916	2.509-9.631
Cox multivariate analysis			
MLR	0.0147	8.775	1.532-50.273
High *versus* low ATA risk	<0.0001	5.998	3.126-11.505
Intermediate *versus* low ATA risk	<0.0001	4.892	2.492-9.605

Statistical significance was set at *p*<0.05.

NLR: neutrophil-lymphocyte ratio, PLR: platelet-lymphocyte ratio, MLR: monocyte-lymphocyte ratio, TNM: tumor-node-metastasis, ATA: American Thyroid Association, HR: hazard ratio, CI: confidence interval.
